# T cell receptor and B cell receptor exhibit unique signatures in tumor and adjacent non-tumor tissues of hepatocellular carcinoma

**DOI:** 10.3389/fimmu.2023.1161417

**Published:** 2023-05-29

**Authors:** Shi Xie, Rong Yan, Anqi Zheng, Mengfen Shi, Longqing Tang, Xueying Li, Jiabang Liu, Yifan Gan, Yu Wang, Deke Jiang, Li Liu, Hongkai Wu, Zhanhui Wang

**Affiliations:** ^1^ Guangdong Provincial Key Laboratory of Viral Hepatitis Research, Department of Infectious Diseases and Hepatology Unit, Nanfang Hospital, Southern Medical University, Guangzhou, China; ^2^ Drug Discovery, HRYZ Biotech Co., Shenzhen, China; ^3^ Department of Hepatobiliary Surgery, Nanfang Hospital, Southern Medical University, Guangzhou, China; ^4^ State Key Laboratory of Respiratory Disease, National Clinical Research Center for Respiratory Disease, The First Affiliated Hospital of Guangzhou Medical University, Guangzhou Medical University, Guangzhou, China

**Keywords:** immune repertoire, immunotherapy, tumor microenvironment, T cell receptor, B cell receptor, prognosis

## Abstract

**Background:**

The tumor microenvironment in hepatocellular carcinoma (HCC) is complicated. Tumor-infiltrating T and B cells play a pivotal role in anti-tumor immunity. T cell receptor (TCR) and B cell receptor (BCR) features may reflect the disease-associated antigen response.

**Methods:**

By combining bulk TCR/BCR-sequencing, RNA-sequencing, whole exome-sequencing, and human leukocyte antigen-sequencing, we examined the immune repertoire (IR) features of tumor and adjacent non-tumor tissues obtained from 64 HCC patients.

**Results:**

High IR heterogeneity with weak similarity was discovered between tumor and non-tumor tissues. Higher BCR diversity, richness, and somatic hypermutation (SHM) were found in non-tumor tissues, while TCRα and TCRβ diversity and richness were comparable or higher in tumor. Additionally, lower immune infiltration was found in tumor than non-tumor tissues; the microenvironment in tumor appeared to keep stably inhibited and changed slightly with tumor progression. Moreover, BCR SHM was stronger, whereas TCR/BCR diversity declined with HCC progression. Importantly, we found that higher IR evenness in tumor and lower TCR richness in non-tumor tissues indicated better survival in HCC patients. Collectively, the results revealed that TCR and BCR exhibited distinct features in tumor and non-tumor tissues.

**Conclusions:**

We demonstrated that IR features vary between different tissues of HCC. IR features may represent a biomarker for the diagnosis and treatment of HCC patients, providing references for subsequent immunotherapy research and strategy selection.

## Introduction

Liver cancer is the third leading cause of cancer-related death worldwide; hepatocellular carcinoma (HCC) accounts for 90% of liver cancer (1), with a 5-year survival of 18% (2). Approximately 72% of HCC occurred in Asia, with over 50% reported in China. Hepatitis B virus infection is the leading cause of HCC, followed by alcoholic, aflatoxin, hepatitis C virus infection, and nonalcoholic fatty liver disease (3).

Treatment options for HCC depend on the tumor burden, degree of liver dysfunction, and performance status. Radiofrequency ablation, surgical resection, transplantation, and radioembolization are the main treatment options for very early-stage HCC. Combination therapy is more commonly utilized in early- or intermediate-stage HCC. Regrettably, therapeutic choices for advanced-stage HCC are currently limited. Sorafenib or lenvatinib are used in first-line therapy for advanced-stage HCC; however, their effectiveness is not satisfactory. Although the use of immune checkpoint inhibitor may result in good outcome in melanoma, its overall efficacy in liver cancer remains limited (4). Adoptive cell transfer therapy with high affinity and high recognition ability for tumors (5), combined with immune regulator and neoantigen vaccination, is a potential approach to tumor therapy (6).

The tumor microenvironment (TME) includes immune cells, stromal cells, malignant cells. The TME exhibits high intertumor and intratumor heterogeneity (7, 8). Lower tumor mutation burden (TMB) or tumor neoantigen burden (TNB) has been observed in HCC compared with melanoma and lung carcinoma (9, 10). High TMB or TNB suggests higher sensitivity to immunotherapy and better outcome (11). Loss of heterozygosity (LOH) at a human leukocyte antigen (HLA) locus is an important mechanism for immune evasion by tumor (12, 13). Moreover, the mechanism through which tumor molecular features, (TMB, TNB, purity, ploidy), and immune infiltration influence T cell receptor (TCR) and B cell receptor (BCR) features or their associations remain unknown.

The immune repertoire (IR) includes the TCR repertoire and BCR repertoire. The diversity of both TCR and BCR is mainly composed of variable (V), diversity (D), and joining (J) gene segments. Additional diversity is generated by non-templated nucleotide (N) additions and/or deletions at gene junctional regions (14). Studies conducted thus far on IR have mostly focused on TCR (mainly TRB), ignoring other TCR chains and BCR. Despite the presence of fewer B cells in tumors compared with T cells, tumor-infiltrating B cells play a crucial role in tumor immunity. They can exhibit anti-tumor or pro-tumor activity, or interact with tumor-infiltrating T cells (15). During tumor progression, the role of features of TCR and BCR and their relationships in tumor and adjacent non-tumor tissues remain unknown. Research on TCR and BCR by combining tumor-infiltrating T and B cells may clarify the IR signatures in tumors.

In this study, we examined differences between BCR IgH and TCR in HCC. We also clarified the immune infiltration and their relationship between IR features in tumor and adjacent non-tumor tissues. Furthermore, we analyzed the correlations between IR features and prognosis in HCC patients.

## Materials and methods

### Patient cohort and sample collection

Samples were collected from May 2014 to December 2018 in Nanfang Hospital (Guangzhou China). A total of 64 HCC patients were enrolled in this study. Paired tumor and adjacent non-tumor tissues (at least 2 cm × 2 cm × 2 cm) were obtained from HCC patients during resection surgery. All specimens were frozen in liquid nitrogen or at −80°C. This study was conducted in accordance with the principles of the Declaration of Helsinki and approved by the Institutional Ethics Committee of Nanfang Hospital, approval number NFEC-201208-K3. All patients provided written informed consent.

### High-throughput sequencing

Total RNA was isolated from tissues using TRIzol reagent (Invitrogen, Carlsbad, CA, USA). For the TCR/BCR library construction, 5’ rapid amplification of cDNA ends and two-round PCR were performed using specific primers for TCR and BCR IgH amplification as previously described (16). All PCR products were identified on 2% agarose gels, and purified using the Qiaquick Gel Extraction kit (Qiagen, Hilden, Germany). Illumina adaptors (Illumina, San Diego, CA, USA) were ligated using the NEBnext Ultra DNA Library Prep kit (New England Biolabs, Ipswich, MA, USA). Subsequently, all products were subjected to high-throughput sequencing using the Illumina HiSeq3000 platform, with a read length of 2×150 bp.

All tissue samples were sent to Novegene (Beijing, China) for bulk RNA sequencing (RNA-seq), whole exome-sequencing (WES), and HLA sequencing (HLA-seq). Original sequencing data were processed by Novegene.

### IR data processing and analysis

Original data provided by the sequencing company were converted to raw sequence by base calling. Next, the results were sorted in FASTQ format. Low-quality sequences were discarded. The V, D, and J gene identification, CDR3 sequence extraction, and error corrections in clean reads of TCR and BCR were performed using MiXCR (v3.0.13) (17). The productive reads meeting the following conditions were further analyzed: (i) any reads with CDR3 amino acid (CDR3aa) length ≥4; (ii) length in CDR3 nt was a multiple of 3; (iii) CDR3 nt sequences did not contain stop codons. The richness of one CDR3aa or VDJ repertoire was evaluated based on the number of unique clonotypes. The evenness of one CDR3aa or VDJ repertoire was evaluated with the normalized Shannon diversity entropy (NSDE). Diversity discussed in this study included richness and evenness. The similarity of two CDR3aa or VDJ profiles was evaluated using the Morisita–Horn similarity index (MHSI). All IR features are shown as the CDR3aa and VDJ level.

BCR mutated clone average degree was calculated as follows. Each mutated clone in each sample was considered as a node; the nodes were connected to construct a mutated clone net for each sample. The mutated clone average degree is equal to the number of connections each node has with other nodes, divided by the total number of nodes.

### RNA-seq, WES, and HLA-seq data analysis

The bulk RNA-seq data were processed to filter out low-quality reads by “-w 5-| 75 -q 20” using FASTP (v 0.20.0) (18). Differential gene expression was analyzed using egdeR (19). TMB and TNB were determined using Mutect2 (20) and VarScan (21) and NetMHCpan (version 4) (22), respectively. HLA typing and HLA LOH analysis were performed using seq2HLA (23) and LOHHLA (12). Tumor ploidy and purity were analyzed with Sequenza (24). The immune score and immune cell infiltration were analyzed by CIBERSORT (25). Gene enrichment analysis was processed by Kyoto Encyclopedia of Gene and Genomes (KEGG) (26) and Gene Ontology (GO) (27).

### Statistical analysis

The Wilcoxon signed-rank test and the Mann–Whitney *U*-test were used for comparisons between two groups. The Wilcoxon rank-sum test was used to compared independent samples of tumor or non-tumor tissues. The Spearman’s rank order correlation coefficient was used to calculate correlations. The χ^2^ or Fisher’s exact tests were used to analyze categorial variables. Continuous variables were expressed as the mean ± standard deviation or median and interquartile range. Two-side *p*-values <0.05 denoted statistically significant differences. The SPSS software version 24 (IBM Corporation, Armonk, NY, USA) was used for the statistical analysis.

## Results

### Patient cohort and characteristics of TCR/BCR sequences

**Figure 1:**
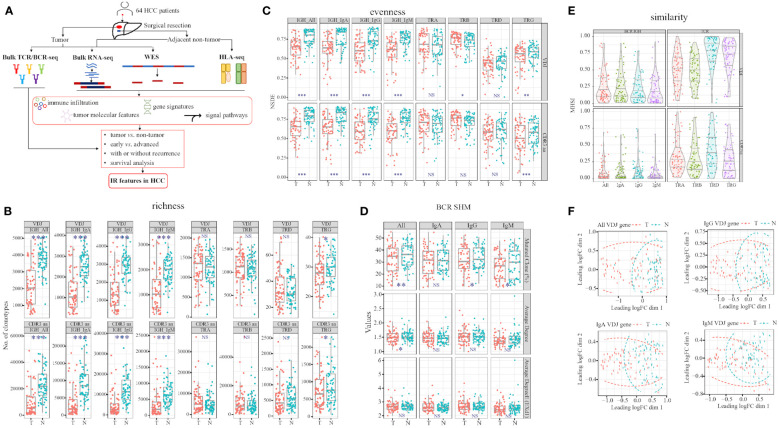
Workflow and TCR and BCR features differ in tumor and adjacent non-tumor tissues. **(A)** Study workflow. Comparison of richness **(B)** and evenness **(C)** in tumor and adjacent non-tumor tissues in HCC patients. **(D)** Three values in BCR somatic hypermutation (SHM). **(E)** Comparison of MHSI value between tumor and adjacent non-tumor tissues in TCR and BCR. **(F)** BCR VDJ gene PCA clustering. T, tumor; N, adjacent non-tumor tissues. Left panel: BCR; right panel: TCR. *p<0.05, **p<0.01, ***p<0.001, according to Wilcoxon signed-rank test. BCR, B cell receptor; HCC, hepatocellular carcinoma; IR, immune repertoire; WES, whole exome sequencing; HLA, human leukocyte antigen; MHSI, Morisita–Horn similarity index; TCR, T cell receptor; Average degree (EXd1), mutated clone average degree removed node with connection only 1; PCA, principal component analysis. NS, no significance.

In this study, we enrolled 64 patients with pathologically diagnosed HCC to analyze the differences in the TCR repertoire and BCR IgH repertoire, as well as their associations with outcome in HCC patients. For a better analysis of the IR features in HCC, TNM stage 1 was defined as early-stage disease, while TNM stage 2 and above were defined as advanced-stage disease. The number of patients with early- and advanced-stage disease was 31 and 33, respectively. The major clinical information of patients is presented in **Table 1**. All patients were treatment naïve and first diagnosed with HCC during resection surgery. IR-seq, RNA-seq, WES, and HLA-seq were combined for analysis (**Figure 1A**). There was no significantly difference found in sequences reads for TRA, TRB, and BCR between tumor and non-tumor tissues (**Supplementary Figure 1**).

**Table 1:** Major clinical information of patients.

Characteristics	Cases	Frequency
Total patients	64	
Sex		
Male	55	85.9%
Female	9	14.1%
Age		
18 1 40y	11	17.2%
40~60y	39	60.9%
60y	14	21.9%
HBV-related		
Yes	59	92.2%
No	5	7.8%
Stage		
Early	31	48.4%
Advanced	33	51.6%
Grade		
High/Mediate-High	11	17.2%
Mediate	46	71.9%
Others	7	10.9%
Vascular invasion		
Yes	21	32.8%
No	43	67.2%
Recurrence/Metastatic		
Yes	35	54.7%
No	24	37.5%
Unknown	5	7.8%
Status		
Alive	41	64.1%
Died	17	26.6%
Censored	6	9.3%

### TCR and BCR signatures differed in HCC patients

After filtering all the sequences using MixCR, we analyzed the TCR and BCR features in HCC patients. We discovered that both richness and evenness of BCR and TRG were significantly higher in non-tumor than tumor (**Figure 1B**). However, in TCR, TRB VDJ evenness were significantly higher in tumor than non-tumor (**Figure 1C**; **Supplementary Tables 1**, **2**). Furthermore, we calculated the BCR somatic hypermutation (SHM) in paired tissues (28), and found higher percentages of IgG and IgM mutated clones in non-tumor than tumor tissues (**Figure 1D**).

These findings suggest that BCR IgH and TRG clonotypes are distributed more abundantly and evenly in non-tumor tissues, in contrast to TRB diversity. In addition, higher BCR SHM indicated sustained and durable B cell response in non-tumor tissues. In summary, TCR and BCR exhibited different signatures between tumor and non-tumor tissues of HCC.

### Stronger similarity and overlap between tumor and non-tumor tissues in TCR than BCR

To assess the similarity of TCR/BCR between tumor and non-tumor tissues obtained from each patient, we calculated the MHSI of each paired sample. The strongest and the weakest similarity were observed in TRD and TRB among all TCR, respectively. In BCR, IgM and IgG exhibited the lowest and the highest similarity, respectively (**Figure 1E**; **Supplementary Table 3**). In summary, the MHSI values were all markedly higher in TCR than BCR, indicating that BCR similarity between tumor and non-tumor tissues was weaker than TCR similarity.

Meanwhile, we compared two groups using principal component analysis (PCA) and multi-dimensional scaling (MDS). Tumor and non-tumor tissues can be well defined by MDS only combined BCR VDJ gene, combined all the heavy chain VDJ gene was better than one isotype alone (**Figure 1F**). Other combinations did not distinguish tumor from non-tumor tissues (**Supplementary Figure 2**). Greater dispersion was observed in tumor than non-tumor tissues.

### Correlation between different TCR chains and/or BCR isotypes

**Figure 2:**
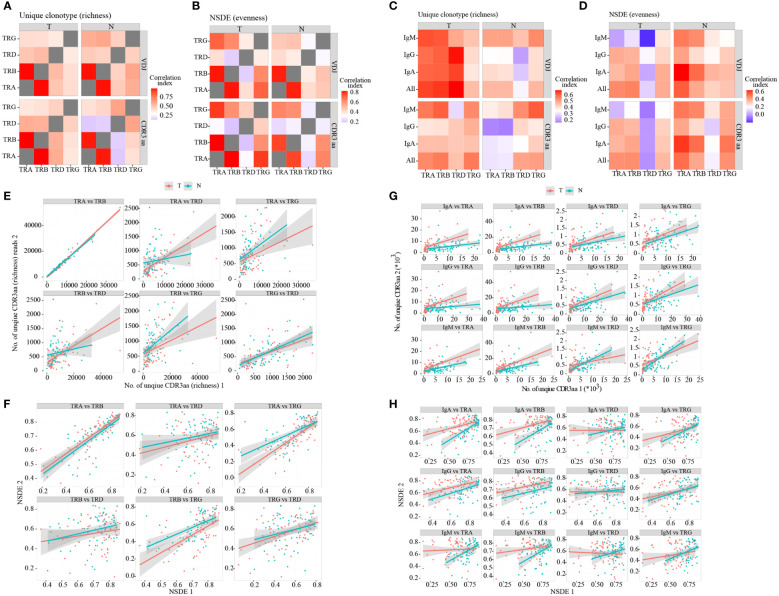
Correlation between TCR chains and BCR isotypes. Heatmap of correlations between TCR different chains in terms of richness **(A)** and evenness **(B)**. Heatmap of correlations between TCR different chains and BCR isotypes in terms of richness **(C)** and evenness **(D)**. The heatmap bar indicates the correlation index. **(E–H)** Dot plot of correlations between TCR and BCR. Red: tumor; green: adjacent non-tumor. BCR, B cell receptor; TCR, T cell receptor.

Considering that neoantigen in the TME may induce T and B cell response. Moreover, B cells can present antigens to T cells, and their interaction can promote each other’s activation. Hence, we analyzed the correlation between richness and evenness in different TCR chains (**Supplementary Figure 3A**; **Supplementary Tables 4**, **5**) and BCR isotypes (**Supplementary Figures 3B, C**; **SupplementaryTables 6**, **7**). We discovered that correlations between TRA and TRB were both higher in richness (**Figures 2A, E**) and evenness (**Figures 2B, F**), while correlations between TRD and other TCR/BCR were lower than others’ (**Figures 2A–D**). However, correlations between TCR and BCR exhibited different signatures in tumor and non-tumor tissues (**Figures 2C, D, G, H**; **Supplementary Figures 3B, C**; **Supplementary Table 6**). Higher richness correlations between TCR and BCR were observed in tumor, with statistical significance only in VDJ level. However, higher evenness correlations were found in non-tumor tissues, with statistical significance only in CDR3aa. Collectively, these findings suggested that TRA and TRB expanded more collaboratively in TCR, whereas TRD expanded less collaboratively with others. In the TME, the change between TCR and BCR in richness occurred more simultaneously in tumor tissues, while changes in evenness occurred more simultaneously in non-tumor tissues.

### Less immune infiltration in tumor

**Figure 3:**
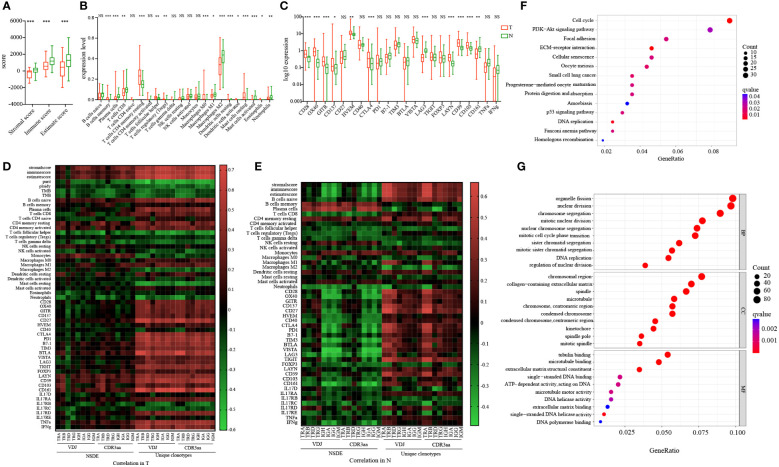
Molecular features and checkpoint expression in tumor and adjacent non-tumor tissues and their connection with IR in HCC patients. **(A)** Immune score in tumor and adjacent non-tumor tissues. **(B)** 22 different types of immune cells infiltration in tumor and adjacent non-tumor tissues. **(C)** Checkpoint expression levels in tumor and adjacent non-tumor tissues. Blue: tumor; green: adjacent non-tumor. *p<0.05, **p<0.01, ***p<0.001, according to Wilcoxon signed-rank test. **(D)** Correlation between immune infiltration and molecular features in tumors of HCC patients. **(E)** Correlation between immune infiltration and molecular features in adjacent non-tumor tissues obtained from HCC patients. The heatmap bar indicates the correlation index. Gene set enrichment analysis based on KEGG **(F)** and GO **(G)**. The size of the circle indicates counts; the color indicates false-discovery rate adjusted *p*-value (q value). GO, Gene Ontology; HCC, hepatocellular carcinoma; IR, immune repertoire; KEGG, Kyoto Encyclopedia of Gene and Genomes. NS, no significance.

IR features differed in tumor and non-tumor tissues, and these features may be affected by elements in the TME. To further investigate the possible mechanism involved in this process, we combined RNA-seq data to analyze the expression of some checkpoints and immune infiltration in paired tissues. The immune score was significantly higher in non-tumor than tumor (**Figure 3A**), indicating more immune infiltration in non-tumor. Next we found that both tumor and non-tumor tissues were infiltrated mainly by macrophages M2 and CD4 memory resting T cells, T cells follicular helper (Tfh) and Tregs expressed higher in tumor while CD8 expressed higher in non-tumor (**Figure 3B**; **Supplementary Figures 4C, D**). In addition, we found that CD28, OX40, GITR, CD137, HVEM, CTLA4, LAYN, CD39 and CD103 were highly expressed in tumor, whereas only LAG3 and CD161 exhibited higher levels in non-tumor tissues (**Figure 3C**). These findings suggested intra-tumoral microenvironment was more inhibited. Based on this evidence, the TME was inhibited and complex, potentially affecting the anti-tumor response.

To further investigate the relationship between IR and immune infiltration, we analyzed the correlations between IR features and immune score, immune cell infiltration, and checkpoint expression (**Figures 3D, E**; **Supplementary Figure 4A**; **Supplementary Table 8**). IR richness was significantly positively related with the immune score, whereas it was negatively related with tumor purity, ploidy, TMB, and TNB. The relationship between IR evenness and immune score was weak and differed in paired tissues. Moreover, in non-tumor tissues, the correlations between naive B cell abundance and IR features were positive in richness and negative in evenness. The relationships between memory B cell and plasma cell abundance and IR features yielded opposite findings. Similar results were found for the correlations between IR features and checkpoints. Collectively, these results suggested that immune infiltration may have different influences in IR richness and evenness in tumor and non-tumor tissues.

Both KEGG (**Figure 3F**) and GO (**Figure 3G**) analyses were conducted to analyze the signaling pathway metabolism in tumor. We discovered that there was no abnormal regulation of immune-related pathways. However, the DNA replication and ATP-dependent activity related genes were enriched in tumor. Our findings indicated that influences on TCR and BCR features may be complex and attributed to multiple reasons, expansion of TCR and BCR in the suppressive microenvironment would be relatively compromised.

### Weak relationship between IR features and tumor clinical characteristics

We also analyzed the correlation between IR features and clinical features. We found that there was weak correlation between most clinical variables and IR features (**Supplementary Figure 5A**). More variables were statistically correlated in terms of evenness, especially in different stages of HCC. TRA and TRB richness in non-tumor tissues was significantly correlated with sex; they were less abundant in female patients than male patients. Also, hypertension may likely influence BCR in tumor. Smoking history may affect IgH and IgG evenness in non-tumor tissues. Cancer thrombus was more strongly related with IR evenness than other clinical variables (**Supplementary Figure 5B**). In non-tumor, we found that BCR evenness decreased with the advancing age of HCC patients; however, this finding was not observed in tumor (**Supplementary Figure 5C**). In summary, the relationships between IR features and clinical variables mainly involved evenness, with few differences observed in richness. These results indicated that researchers should pay more attention to evenness analysis when investigating clinical variables.

### Higher TCR/BCR evenness in early-stage HCC

**Figure 4:**
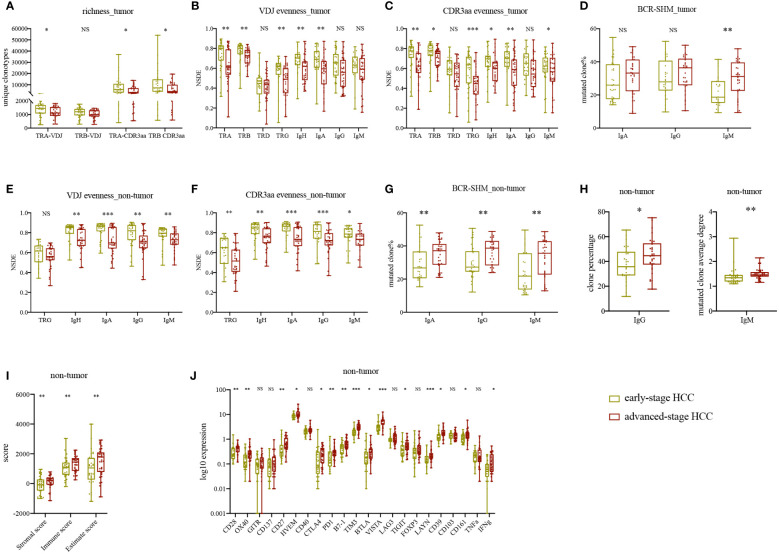
Statistically significant differences in IR and molecular features between early- and advanced-stage HCC. **(A)** TRA and TRB VDJ/CDR3aa richness in tumor tissues. **(B)** IR VDJ evenness in tumor tissues. **(C)** IR CDR3aa evenness in tumor tissues. **(D)** BCR mutated clone percentage in tumor tissue. **(E)** IR VDJ evenness in adjacent non-tumor tissue. **(F)** IR CDR3aa evenness in adjacent non-tumor tissue. **(G)** BCR mutated clone percentage in adjacent non-tumor tissue. **(H)** IgG percentage and IgM average degree in adjacent non-tumor tissue. **(I)** Immune score in adjacent non-tumor tissue. **(J)** Checkpoint and CD markers expression levels in adjacent non-tumor tissue. green: early-stage HCC; red: advanced-stage HCC. *p<0.05, **p<0.01, ***p<0.001, according to Wilcoxon signed-rank test. BCR, B cell receptor; HCC, hepatocellular carcinoma; IR, immune repertoire; SHM, somatic hypermutation; VDJ, variable, diversity, and joining. NS, no significance.

We classified our patients into two groups to identify changes in IR features following progression of HCC. In tumor, we discovered that TRA and TRB CDR3aa richness and evenness, as well as IgH and IgA evenness, were higher in early-stage HCC than advanced-stage HCC (**Figures 4A–D**). Similarly, in non-tumor tissues, TRG and BCR evenness was all higher in early-stage HCC (**Figures 4E, F**). All other IR features in tumor were higher in early-stage HCC, while richness of TRA and TRB in non-tumor tissues was comparably higher in advanced-stage HCC (**Supplementary Figures 6A–C**). The results indicated that TCR and BCR evenness was reduced following HCC progression; however, richness did not change as obviously as evenness.

### Stronger BCR SHM and higher immune score in advanced-stage HCC

Besides that, we analyzed the original clone percentage, mutated clone percentage, and mutated clone average degree in BCR. IgA, IgG, and IgM mutated clone percentages were higher in tumor tissues of advanced-stage HCC than early-stage HCC; nevertheless, only IgM presented statistically significant results (**Figure 4D**). In non-tumor tissues, the percentages of IgA, IgG and IgM mutated clones, IgG clone percentage, and IgM mutated clone average degree were significantly higher in advanced-stage HCC (**Figures 4G, H**). Other BCR SHM features were also higher in advanced-stage HCC, in contrast to their original clone percentages (**Supplementary Figures 6D, E**). Therefore, as HCC progressed, the IgG clone percentage and BCR mutation load were increased, whereas IgM was decreased, indicating that B cells were under antigen stimulation and differentiation. These findings suggested local class switch recombination and SHM in advanced-stage HCC.

Next, we compared the immune infiltration at different stages of HCC. In non-tumor tissues, the immune score was significantly higher in advanced-stage HCC (**Figure 4I**). Meanwhile, in tumor, immune score, ploidy, tumor purity, TMB, and TNB were higher in advanced-stage HCC than early-stage HCC (**Supplementary Figure 7A**). There was no difference in MHSI between the disease stages for both TCR and BCR except TRG (**Supplementary Figure 7B**). In non-tumor tissues, the expression levels of some checkpoints, such as CD28, OX40, CD27, HVEM, CTLA4, PD1, B7-1, TIM3, VISTA, TIGIT, LAYN and CD39 were higher in advanced-stage HCC (**Figure 4J**). Notably, these differences were not found in tumor. Furthermore, in non-tumor tissues, we found that the memory B cells expressed higher in early-stage HCC, neutrophils expressed higher in advanced-stage HCC, there were no significant differences in other T or B cells (**Supplementary Figures 7C, D**) and HLA LOH (**Supplementary Figure 7E**) between early- and advanced-stage HCC in paired tissues. Ultimately, in advanced-stage HCC, there was less immune infiltration and more inhibition of the TME in tumor than in non-tumor tissues. In summary, unlike the microenvironment in non-tumor tissues, the TME appeared to keep stably inhibited and changed slightly during HCC progression.

### Higher TCR richness in non-tumor tissues and lower IR evenness in tumor tissues of patients with disease recurrence

**Figure 5:**
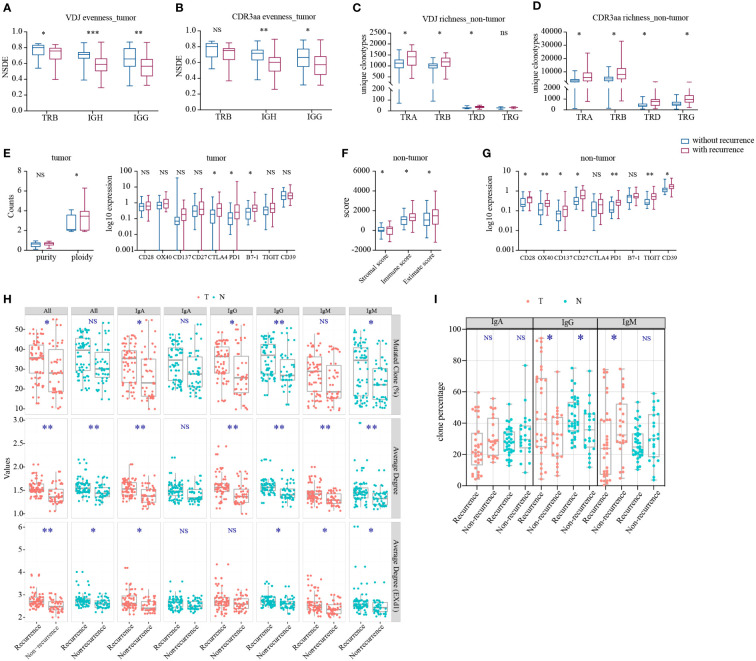
Statistically significant differences in IR and molecular features between HCC patients with and without recurrence. **(A)** IR VDJ evenness in tumor. **(B)** IR CDR3aa evenness in tumor. **(C)** IR VDJ richness in non-tumor tissues. **(D)** IR CDR3aa richness in non-tumor tissues. **(E)** Purity and ploidy, checkpoints expression in tumor. **(F)** Immune score in adjacent non-tumor tissues. **(G)** Checkpoint expression in adjacent non-tumor tissues. blue: without recurrence; red: with recurrence. **(H)** BCR SHM. **(I)** BCR original clonotype percentages in patients with and without recurrence. green: non-tumor tissues; red: tumor. *p<0.05, **p<0.01, ***p<0.001, according to Wilcoxon signed-rank test. BCR, B cell receptor; HCC, hepatocellular carcinoma; IR, immune repertoire; SHM, somatic hypermutation; VDJ, variable, diversity, and joining; Average degree (EXd1), mutated clone average degree removed node with connection only 1. NS, no significance.

Subsequently, we compared the IR features in tumor and non-tumor tissues of HCC patients with or without recurrence or metastasis. Patients with recurrence had significantly lower TRB VDJ, IGH, and IgG (**Figures 5A, B**) evenness in tumor than that in patients without recurrence. Conversely, significantly higher TCR richness was found in patients with recurrence in non-tumor (**Figures 5C, D**). There was no difference in remaining IR features between patients with and without recurrence (**Supplementary Figures 8A–D**). The higher IR evenness in tumor and lower TCR richness in non-tumor tissues may indicate better outcome.

Thereafter, we analyzed BCR SHM in patients with or without recurrence. There was no difference in TMB and TNB between patients with and without recurrence (**Supplementary Figure 8G**). Also, there was stronger BCR SHM in patients with recurrence versus those without recurrence (**Figure 5H**), particularly IgH and IgA in tumor tissues, as well as IgG and IgM in non-tumor tissues. Furthermore, we discovered that the IgG clone percentage was higher in patients with recurrence, but that of IgM in tumor tissues was lower (**Figure 5I**). With tumor progression, the BCR SHM burden and the BCR class switch recombination in tumor were increased.

Next, we compared the immune infiltration in patients with or without recurrence. In tumor, we found that the expression of tumor ploidy, CTLA4, PD1, and B7-1 was significantly higher in patients with recurrence versus those without recurrence (**Figure 5E**), but there was no statistical significance found in immune score (**Supplementary Figure 8H**). In non-tumor tissues, immune score and the expression of CD28, OX40, CD137, CD27, PD1, TIGIT, and CD39 were significantly higher in patients with recurrence than those without recurrence (**Figures 5F, G**). However, there was no significant difference found in other markers and HLA LOH (**Supplementary Figures 8F, I**). These findings indicated that, despite the inhibited environment in tumor, there was more immune infiltration in non-tumor tissue from patients with recurrence.

### Lower richness and higher evenness in non-tumor tissues were related with better survival

**Figure 6:**
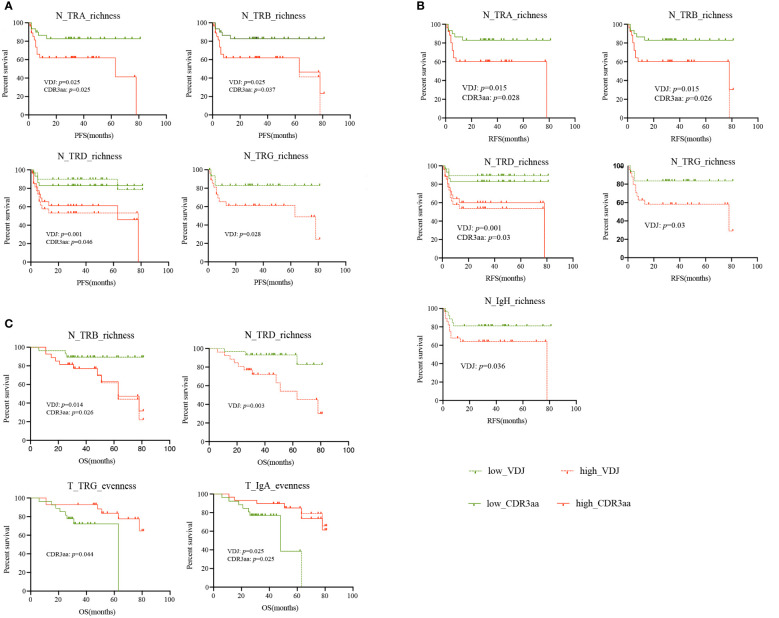
Survival of HCC patients with different IR features. **(A)** PFS. **(B)** RFS. **(C)** OS. green: low IR features; red: high IR features; solid line: CDR3aa; dashed line: VDJ. HCC, hepatocellular carcinoma; IR, immune repertoire; OS, overall survival; PFS, progression-free survival; RFS, recurrence-free survival.

As the TCR and BCR features differed in HCC patients, we hypothesized that they may be biomarkers for HCC patients. Therefore, we analyzed progression-free survival (PFS), recurrence-free survival (RFS), and overall survival (OS) in this HCC cohort. We found that patients with lower TRA, TRB, TRD and TRG richness in non-tumor tissues had a longer PFS (**Figure 6A**) and RFS (**Figure 6B**). Similarly, Lower IgH-VDJ richness in non-tumor tissues was linked to better RFS (**Figure 6B**). For OS, we noted that TRB and TRD richness in non-tumor tissues was also significantly lower in patients with longer OS. Patients with higher IgA evenness and TRG CDR3aa evenness in non-tumor tissues had longer OS (**Figure 6C**). Moreover, lower tumor ploidy was found in patients with longer RFS. There was no connection found between other tumor molecular features or HLA LOH and PFS, RFS, and OS (**Supplementary Figure 9**). In summary, lower richness and higher evenness in non-tumor tissues indicated better prognosis in HCC patients.

## Discussion

This study focused on IR in HCC patients. We combined IR-seq, RNA-seq, WES, and HLA-seq to investigate the characteristics of tumor-infiltrating T and B cells. We aimed to clarify the features of TCR and BCR, and identify an IR marker for the diagnosis and treatment of HCC. Studies have shown that tumor-infiltrating T cells are mainly exhausted phenotypes, with high expression of PD1, CD39, CLTA4, TIGIT, or LAG3 (29–31). The CD103, PD1, and CD137 have been considered markers of activated T cells in tumor (32–34). T cells expressing these markers possess more tumor-specific properties, and their TCR may be an alternative for TCR-T therapy. The role of B cells in anti-tumor immunity may be underestimated and controversial. Also, it is unknown whether the TCR and BCR signatures in HCC are similar. Numerous studies have shown high intratumor heterogeneity in clear cell renal cell carcinoma, lung cancer, and HCC (35–37). Neoantigen typically leads to intratumor heterogeneity, which induced different T or B cell anti-tumor immunity, and heterogeneity of TCR and BCR. Research on lung cancer showed that spatial differences of TCR in different regions were driven by different neoantigens (38). Single-cell sequencing is useful for analyzing tumor heterogeneity. However, both the number of cells and patients enrolled are very limited, and different regions may yield varied results. Therefore, we analyzed the TCR and BCR richness, evenness, and similarity in tumor and non-tumor tissues of HCC patients by bulk IR-seq to examine IR features more comprehensively in HCC.

We demonstrated that IR features may differ between tumor and non-tumor tissues. Firstly, BCR IgH exhibited higher richness and evenness in non-tumor tissues. Studies on other tumors also yielded similar results, showing that BCR diversity was lower in tumor than non-tumor, with an overlap <10% (39). In contrast, TCR richness did not differ between tumor and non-tumor tissues. However, TRA and TRB had a relatively higher evenness in tumor, in agreement with our previous study (16). Secondly, we found out that there was low similarity of BCR repertoire between tumor and adjacent non-tumor tissues. The TCR repertoire similarity was slightly higher than that of BCR, indicating that weak clonotypes overlap in tumor and adjacent non-tumor. Thirdly, change in richness between TCR and BCR occurred more simultaneously in tumor, while change in evenness occurred more simultaneously in adjacent non-tumor tissues. Moreover, we focused on B cells in this study. In triple-negative breast cancer, tumor-infiltrating B cells may have a unique antibody repertoire; BCR diversity was lower in tumors, whereas SHM was high (40). Thus, we compared the BCR SHM between tumor and non-tumor tissues, and found higher BCR SHM in the latter type. Subsequently, PCA clustering revealed that the BCR VDJ gene combinations can distinguish tumor and non-tumor tissues, particularly the IgH VDJ gene combination.

TCR and BCR in tumor and adjacent non-tumor tissues exhibited completely different signatures. This is attributed to different roles of TCR and BCR, and the involvement of the TME. The T cells, particularly cytotoxic T lymphocytes, are the pioneers in anti-tumor reactions. They recognized neoantigens or tumor associated antigens, and kill tumor cells directly or by secreting cytokines. B cells mostly kill tumor cells by secreting antibody or inducing antibody-dependent-cell-mediated cytotoxicity to react. Repertoire measures may facilitate our understanding of antigen-driven B cell response in cancer.

To clarify the aforementioned differences, we compared the immune infiltration in tumor and non-tumor tissues. Total immune infiltration was significantly higher in non-tumor than tumor. The type of immune cells in tumor and non-tumor tissues were similar. Studies focusing on HCC showed that M2 macrophages were the main immune cells in tumor (37, 41). That tumor-associated macrophages infiltration may suppress CD8+ T cells infiltration, which further induced the state of immune cells towards a more immunosuppressive and exhausted status (42). It was negatively related with survival because it can promote tumor progression. Thus, activated immune infiltration appeared to be slightly higher in non-tumor tissues, while immune infiltration was more complicated in tumor. Nevertheless, immune infiltration cannot totally reflect the actual immune reaction. Research on esophageal squamous cell carcinoma showed that higher immune score indicated higher TCR diversity in tumor (43). However, the relationship between immune score and IR diversity remains unknown.

We further analyzed the relationship between immune infiltration and IR features. The immune score was positively related with IR richness, and negatively related with the molecular features of tumors (**Supplementary Figure 4B**). Similarly, checkpoint expression was positively related with IR richness in paired tissues. We hypothesized that richness may be less affected by others. Nonetheless, greater diversity was linked to more immune infiltration in tumor, but not in non-tumor tissues. We also observed that correlations between BCR signatures and some checkpoint expression differed in paired tissues. In tumors, increased expression of those checkpoints may be associated with a more diverse BCR repertoire. However, such connection wasn’t found in non-tumor tissues. These results suggested the presence of a more complicated microenvironment in tumors. Therefore, using a single metric may not be sufficient for the accurate evaluation of the microenvironment in HCC.

We analyzed the TCR and BCR features at different stages of HCC. IR diversity declined while the BCR SHM and mutated clone percentages were increased with the progression of HCC. Thus, as tumors grow, the BCR may be under markedly more selection pressure and experience more antigen stimulation. This process leads to more SHM in B cells (44) and potentially higher affinity after several rounds of SHM (39). The presence and number of neoantigen-related B cells contained warrants further investigation (45). Moreover, a study on HCC demonstrated that the expression of some inhibited checkpoints was upregulated in tumor, and this effect was related with a more exhausted microenvironment (46). Although IR diversity was lower, immune infiltration in non-tumor tissues was increased with HCC progression. One of the hypothesis is that these immune elements were attracted by tumor signals, but are unable to enter the tumor. The immune cells appeared to gather around the tumors and mostly accumulated in non-tumor tissues. The reasons for this inability of immune cells remain to be determined. A reason may be the presence of a barrier between tumor and non-tumor tissues. A study investigating spatial architecture in primary liver cancer revealed that the tumor capsule potentially affects immune cell infiltration and transcriptome diversity (47), further reflecting the IR features. In addition to that, there was the immune barrier structure located near the tumor boundary, which was a spatial niche composed of SPP1+ macrophages and cancer-associated fibroblasts. The tumor immune barrier may limit the immune infiltration in the tumor core (48).

We noticed that while TCR richness in non-tumor tissues was elevated, suppressive CD4+ T cells such as Treg, which are highly expressed in tumor compared with non-tumor tissues, changed less with tumor progression. First, the expression of genes associated with tumor-infiltrating Tregs (46) such as CTLA4, GITR, LAYN and OX40 was significantly higher in tumor tissues compared to non-tumor tissues. Second, it has been shown that Treg is enriched within tumors as a result of several different mechanisms: increased infiltration, local expansion, survival advantage, and *in situ* development from conventional CD4+ T cells (49). There are also combined Treg TCR and transcriptome sequencing results suggesting that intra-tumor Treg enrichment is the result of *in situ* amplification, with clonal Treg TCRs within tumors are 2-fold higher than paraneoplastic ones, whereas clonal Treg TCRs can be 4-5-fold higher than paraneoplastic ones (46, 50). Moreover, increased mitochondrial metabolism and oxidation-related metabolism within the tumor tissue, combined with increased neutrophil expression within the tumor, which we could also find in this study, will synergize again to naive T to Treg conversion. Increased Treg expression within tumors is a result of local antigen-driven selection and environmental induction.

Studies on other tumors analyzed the relationship between TCR diversity and clinical outcome, and yielded complex results. T cell function and abundance may differ in tumors, and TCR sequences can reflect dynamic change in T cells (8). In non-small cell lung cancer, greater numbers of T cells and higher clonality of TCR were positively associated with OS (9). However, there is limited research on the relationship between BCR diversity and prognosis. In the present study, higher IgA evenness and lower IgH richness in non-tumor tissues were related with longer OS and PFS. HLA loss is a potential mechanism of immune escape, and higher TMB and lower tumor purity may be related to longer OS (51). Nevertheless, we did not observe such results in our study.

There were some limitations in this study. Firstly, this investigation was mainly based on bulk sequencing, which cannot accurately distinguish the immune cells in tumor and non-tumor tissues. Secondly, we could not identify the functional TCR or BCR for the treatment of tumors. Finally, we have not examined in detail the relationship between IR and tumors. Thus, further investigation is warranted.

In conclusion, this study provides a landscape for IR features in HCC. We demonstrated the IR features and their relationship with immune infiltration and molecular signature by combining bulk TCR/BCR sequencing, bulk RNA-seq, WES, and HLA-seq. We discovered that IR features varied with HCC progression, though some were consistent with others. Furthermore, we found that TCR richness in non-tumor tissues may be used as potential indicators for predicting the prognosis of HCC. The present findings may serve as a basis for further studies of particular T or B cells, as well as the development of novel immunotherapeutic strategies by combining single-cell sequencing, cell phenotyping, expression profiling, and functional analysis.

## Data availability statement

The raw sequence data reported in this study have been deposited in the Genome Sequence Archive (Genomics, Proteomics & Bioinformatics 2021) in National Genomics Data Center (Nucleic Acid Res 2022), China National Center for Bioinformation / Beijing Institute of Genomics, Chinese Academy of Sciences (GSA-Human: HRA004388) that are publicly accessible at https://ngdc.cncb.ac.cn/gsa-human.

## Ethics statement

The studies involving human participants were reviewed and approved by the Institutional Ethics Committee of Nanfang Hospital. The patients/participants provided their written informed consent to participate in this study.

## Author contributions

ZW, LL, DJ, and SX conceived and designed the study. SX, RY, and AZ performed the experiments. SX, RY, AZ, MS, JL, YG, XL, and YW collected samples and clinical information. SX, RY, LT, and HW performed the computational analyses. ZW, SX, RY, and HW interpreted the data. ZW and SX wrote and revised the paper. All authors contributed to the article and approved the submitted version.

## Supplementary material

Supplementary Figure 1 Match reads from sequence data for each patient. Results based on TCR or BCR index in expansion. **(A)** TCR-alpha and TCR-beta clone percentage. **(B)** BCR clone percentage. Top panel: tumor tissue; bottom panel: adjacent non-tumor tissue. BCR, B cell receptor; TCR, T cell receptor.

Supplementary Figure 2 Cluster analysis for VDJ gene in TCR and BCR. MDS for clonotype abundance clustering in TCR **(A)** and BCR **(B)**. PCA for clonotype frequency cluster in TCR **(C)** and BCR **(D)**. BCR, B cell receptor; TCR, T cell receptor; MDS, multi-dimensional scaling; PCA, principal component analysis; VDJ, variable, diversity, and joining.

Supplementary Figure 3 Boxplot for the correlation between TCR chains and BCR isotypes. **(A)** Different TCR chains. **(B)** TCR chains versus BCR isotypes. **(C)** BCR isotypes versus TCR chains. Red: tumor tissue; green: adjacent non-tumor tissue. BCR, B cell receptor; TCR, T cell receptor.

Supplementary Figure 4 Correlation between immune cell infiltration and tumor molecular features in tumor and adjacent non-tumor tissues. **(A)** Correlation between immune score and TRB CDR3aa richness and IgA CDR3aa evenness in tumor tissues, and IgA CDR3aa evenness in adjacent non-tumor tissues. **(B)** Correlations between scores and molecular features of tumors. Circle size and number in it indicate the correlation index. Immune cell infiltration in tumor tissues **(C)** and adjacent non-tumor tissues **(D)**. TRB, T cell receptor-beta.

Supplementary Figure 5 Immune repertoire and clinical variables. **(A)** Range of correlation index and *p*-value for IR and clinical variables. **(B)** Correlation between IR and categorical clinical variables in terms of richness and evenness. **(C)** Correlation between IR and continuous clinical variables in terms of richness and evenness. IR, immune repertoire.

Supplementary Figure 6 Differences in IR and BCR SHM between early- and advanced-stage HCC. **(A)** IR richness in tumor tissues. **(B)** IR richness in adjacent non-tumor tissues. **(C)** IR evenness in non-tumor tissues. BCR SHM differences in tumor **(D)** and adjacent non-tumor tissues **(E)**. green: early-stage HCC, red: advanced-stage HCC. BCR, B cell receptor; HCC, hepatocellular carcinoma; IR, immune repertoire; SHM, somatic hypermutation.

Supplementary Figure 7 Difference in immune infiltration between early- and advanced-stage HCC. **(A)** Tumor molecular and stromal score, immune score and estimate score. **(B)** MHSI. Immune cell infiltration in tumor and adjacent non-tumor tissues **(C, D)** green: early-stage HCC, red: advanced-stage HCC. **(E)** Frequency of HLA LOH at different stages of HCC. Left: early-stage HCC, right: advanced-stage HCC; grey: without HLA LOH; black: with HLA LOH. HCC, hepatocellular carcinoma; HLA, human leukocyte antigen; LOH, loss of heterozygosity; MHSI, Morisita–Horn similarity index.

Supplementary Figure 8 Differences in IR and molecular features between HCC patients with and without recurrence. **(A)** IR richness in tumor tissues. **(B)** IR evenness in tumor tissues. **(C)** IR richness in non-tumor tissues. **(D)** IR evenness in non-tumor tissues. **(E)** Immune cells infiltration in tumor and non-tumor tissues. **(F)** Checkpoint expression in tumor and non-tumor tissues. **(G)** Molecular features of tumors. **(H)** Immune score in tumor tissues. blue: without recurrence; red: with recurrence. **(I)** LOH in HLA. Left: without recurrence, right: with recurrence; grey: without HLA LOH; black: with HLA LOH. HCC, hepatocellular carcinoma; HLA, human leukocyte antigen; IR, immune repertoire; LOH, loss of heterozygosity.

Supplementary Figure 9 Survival analysis based on HLA LOH, tumor ploidy, TMB, and TNB in HCC. **(A)** Better survival in patients with HLA LOH. **(B)** Longer RFS in patients with low tumor ploidy. **(C)** No significant difference between high TMB/TNB and low TMB/TNB. HCC, hepatocellular carcinoma; HLA, human leukocyte antigen; LOH, loss of heterozygosity; RFS, recurrence-free survival; TMB, tumor mutation burden; TNB, tumor neoantigen burden.

Supplementary Table 2 Original information for CD markers expression.
