# Supplementary figures and images for: T cell receptor and B cell receptor exhibit unique signatures in tumor and adjacent non-tumor tissues of hepatocellular carcinoma
Source: Front Immunol. 2023 May 29;14:1161417. doi: 10.3389/fimmu.2023.1161417 (PMC10258310; doi:10.3389/fimmu.2023.1161417)

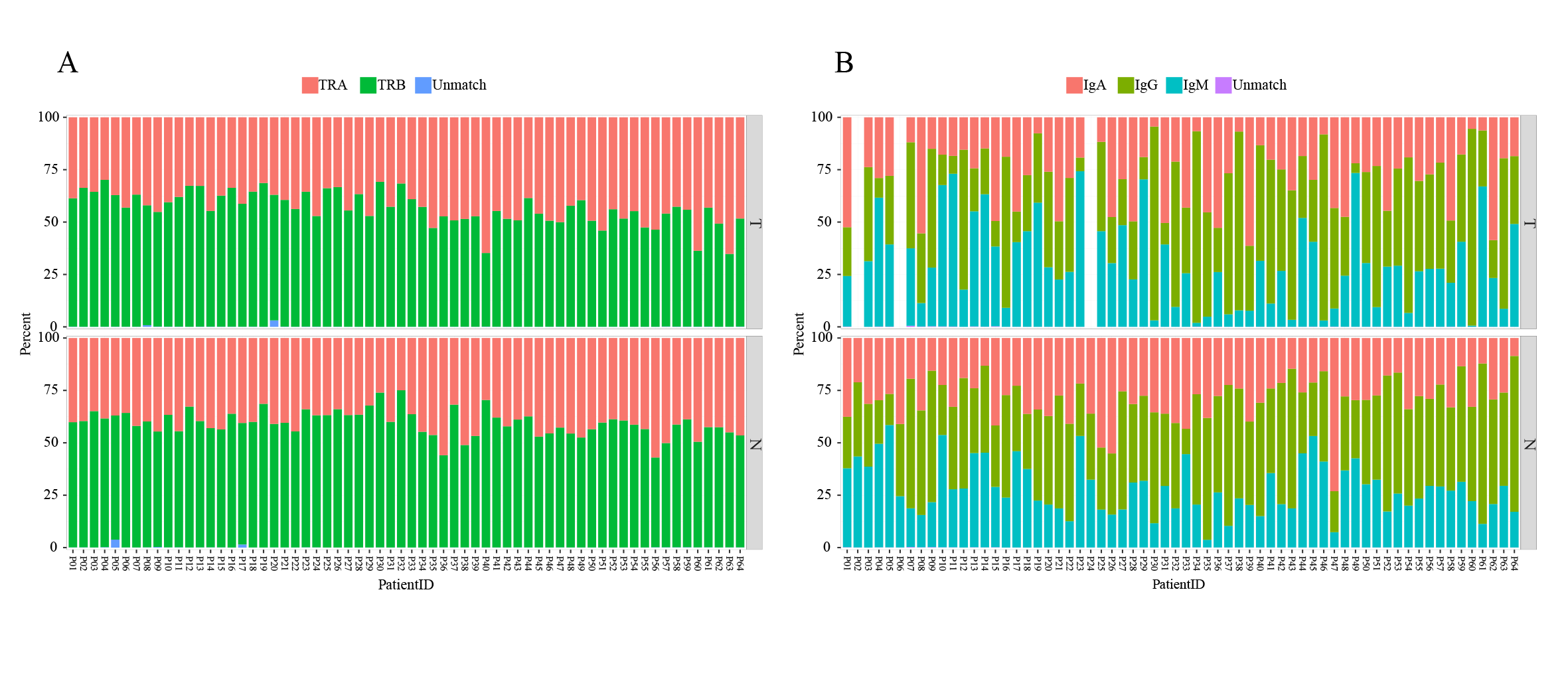

Supplement: Supplementary Figure 1 — Match reads from sequence data for each patient. Results based on TCR or BCR index in expansion. (A) TCR-alpha and TCR-beta clone percentage. (B) BCR clone percentage. Top panel: tumor tissue; bottom panel: adjacent non-tumor tissue. BCR, B cell receptor; TCR, T cell receptor. [file Image_1.tif]

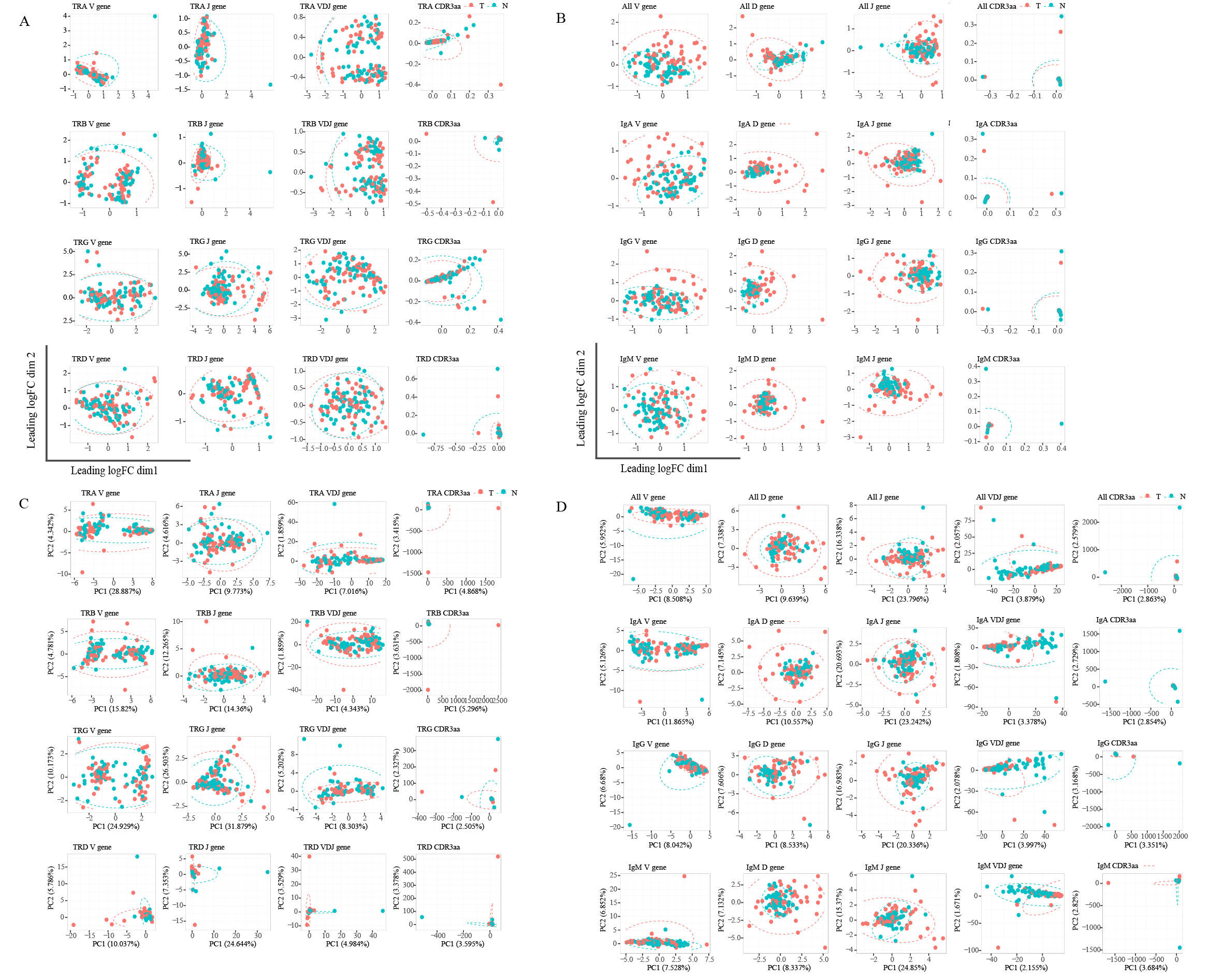

Supplement: Supplementary Figure 2 — Cluster analysis for VDJ gene in TCR and BCR. MDS for clonotype abundance clustering in TCR (A) and BCR (B). PCA for clonotype frequency cluster in TCR (C) and BCR (D). BCR, B cell receptor; TCR, T cell receptor; MDS, multi-dimensional scaling; PCA, principal component analysis; VDJ, variable, diversity, and joining. [file Image_2.tif]

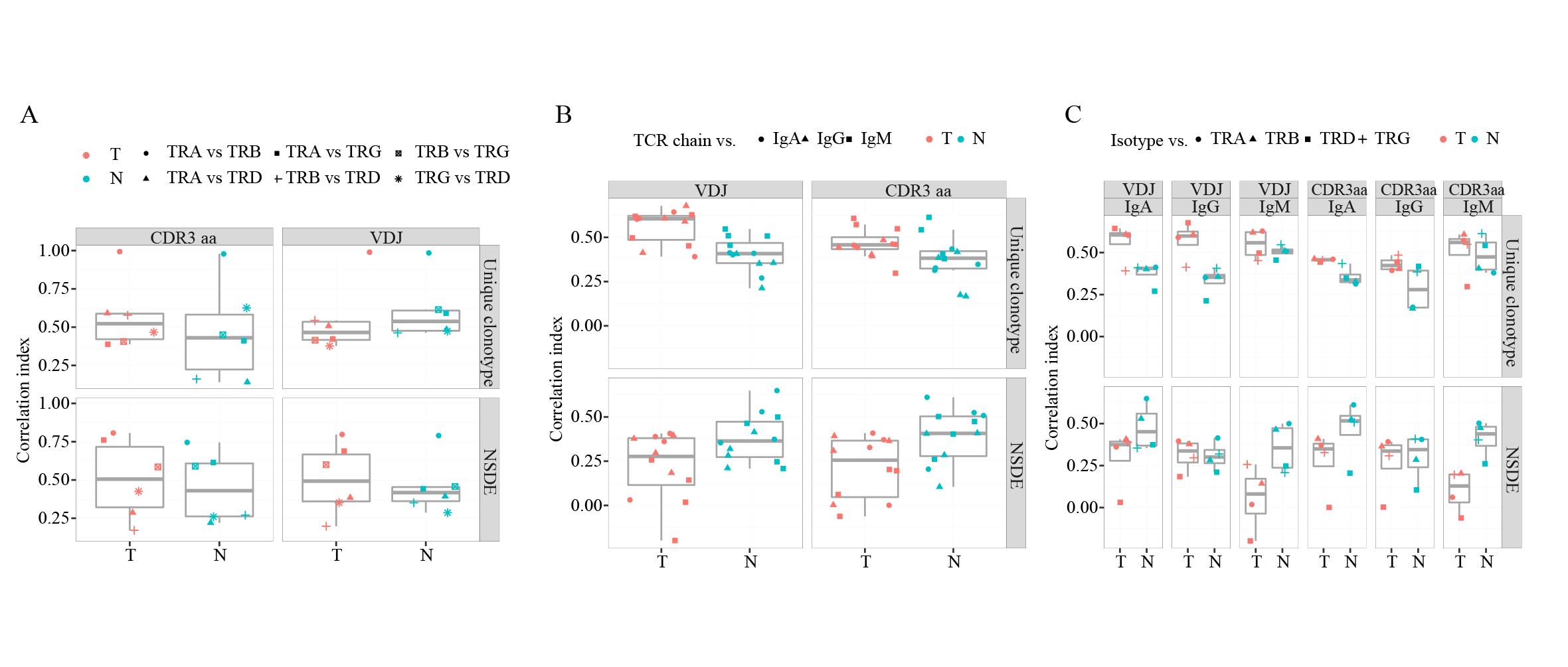

Supplement: Supplementary Figure 3 — Boxplot for the correlation between TCR chains and BCR isotypes. (A) Different TCR chains. (B) TCR chains versus BCR isotypes. (C) BCR isotypes versus TCR chains. Red: tumor tissue; green: adjacent non-tumor tissue. BCR, B cell receptor; TCR, T cell receptor. [file Image_3.tif]

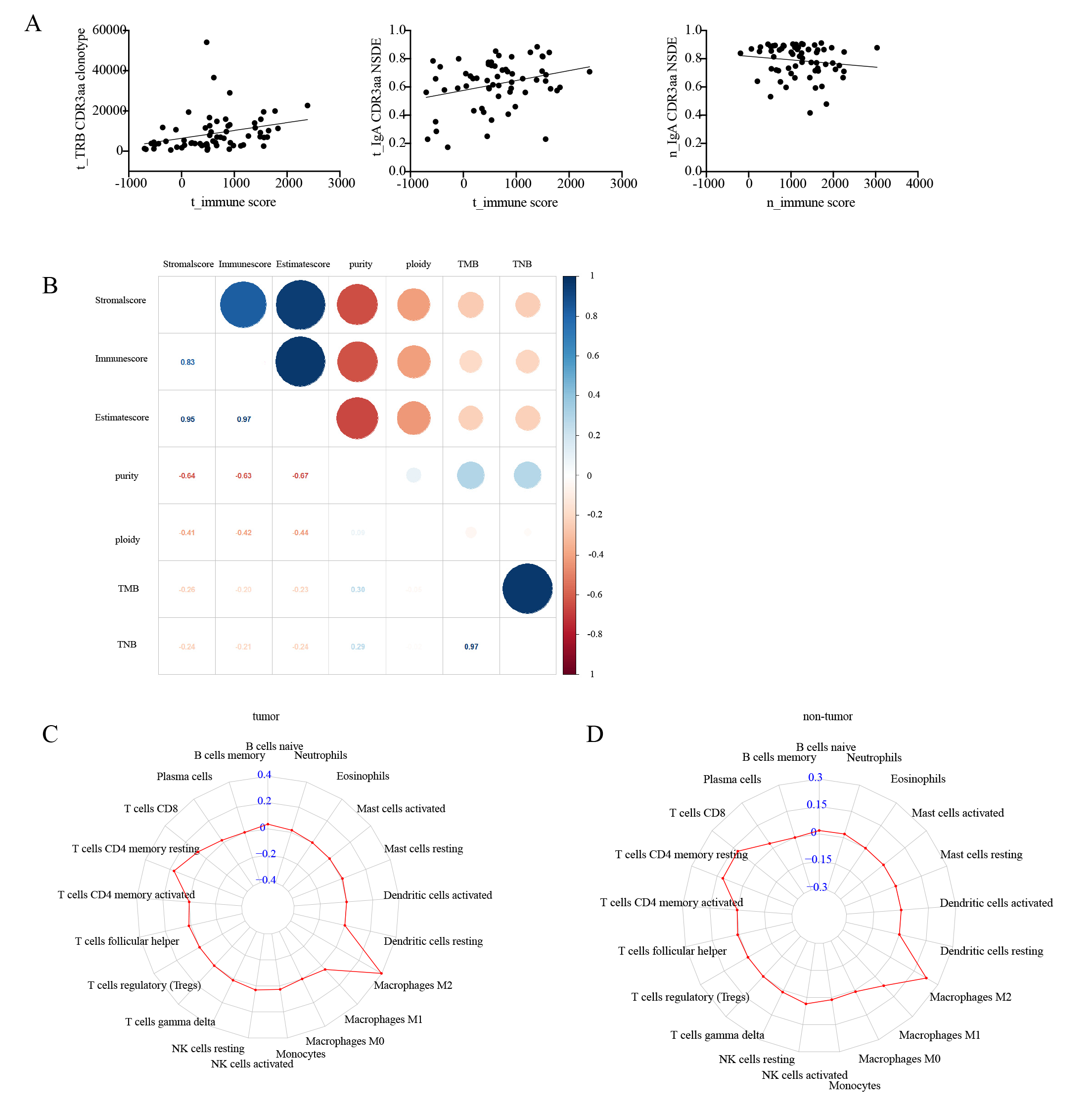

Supplement: Supplementary Figure 4 — Correlation between immune cell infiltration and tumor molecular features in tumor and adjacent non-tumor tissues. (A) Correlation between immune score and TRB CDR3aa richness and IgA CDR3aa evenness in tumor tissues, and IgA CDR3aa evenness in adjacent non-tumor tissues. (B) Correlations between scores and molecular features of tumors. Circle size and number in it indicate the correlation index. Immune cell infiltration in tumor tissues (C) and adjacent non-tumor tissues (D). TRB, T cell receptor-beta. [file Image_4.tif]

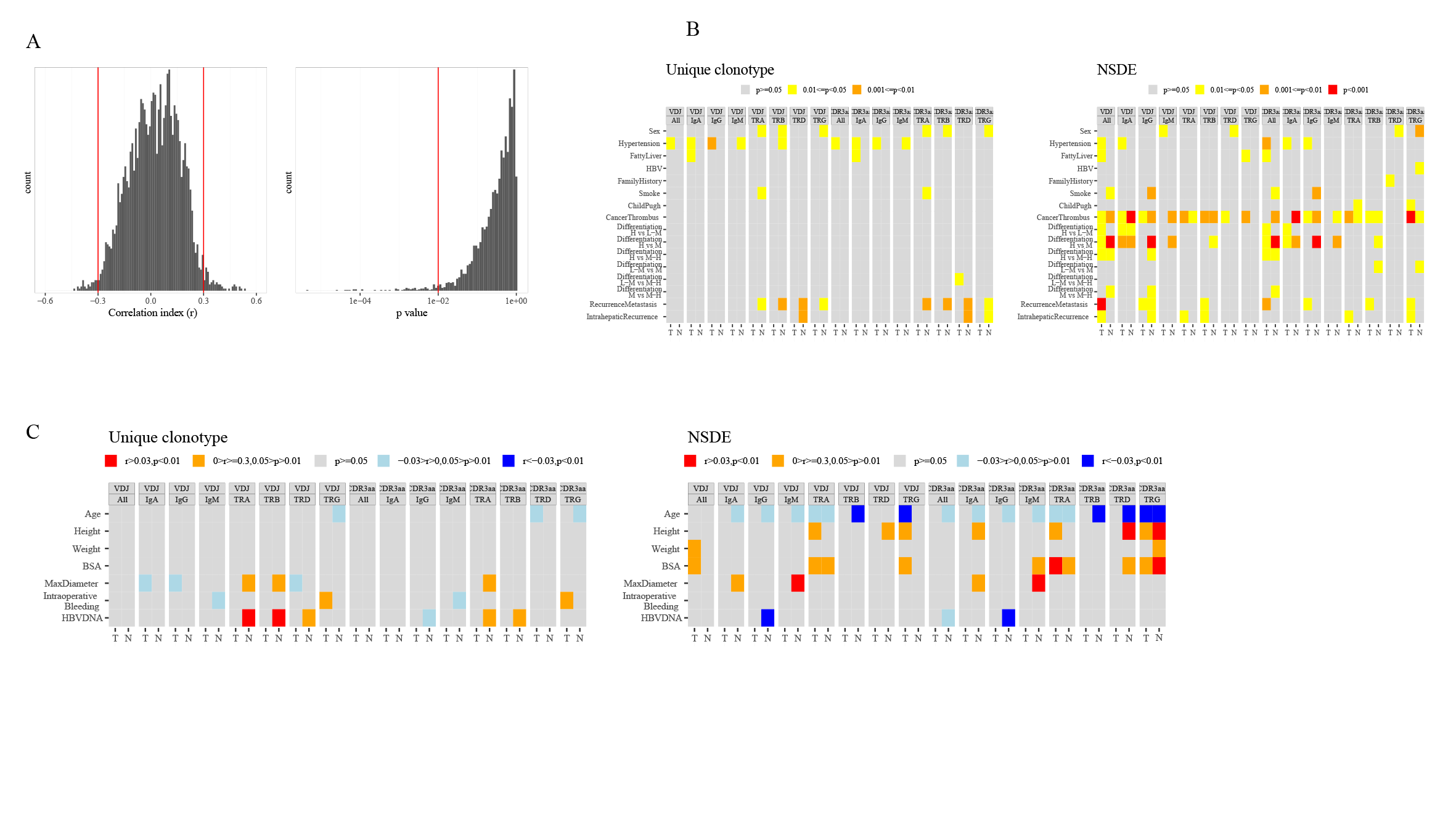

Supplement: Supplementary Figure 5 — Immune repertoire and clinical variables. (A) Range of correlation index and p-value for IR and clinical variables. (B) Correlation between IR and categorical clinical variables in terms of richness and evenness. (C) Correlation between IR and continuous clinical variables in terms of richness and evenness. IR, immune repertoire. [file Image_5.tif]

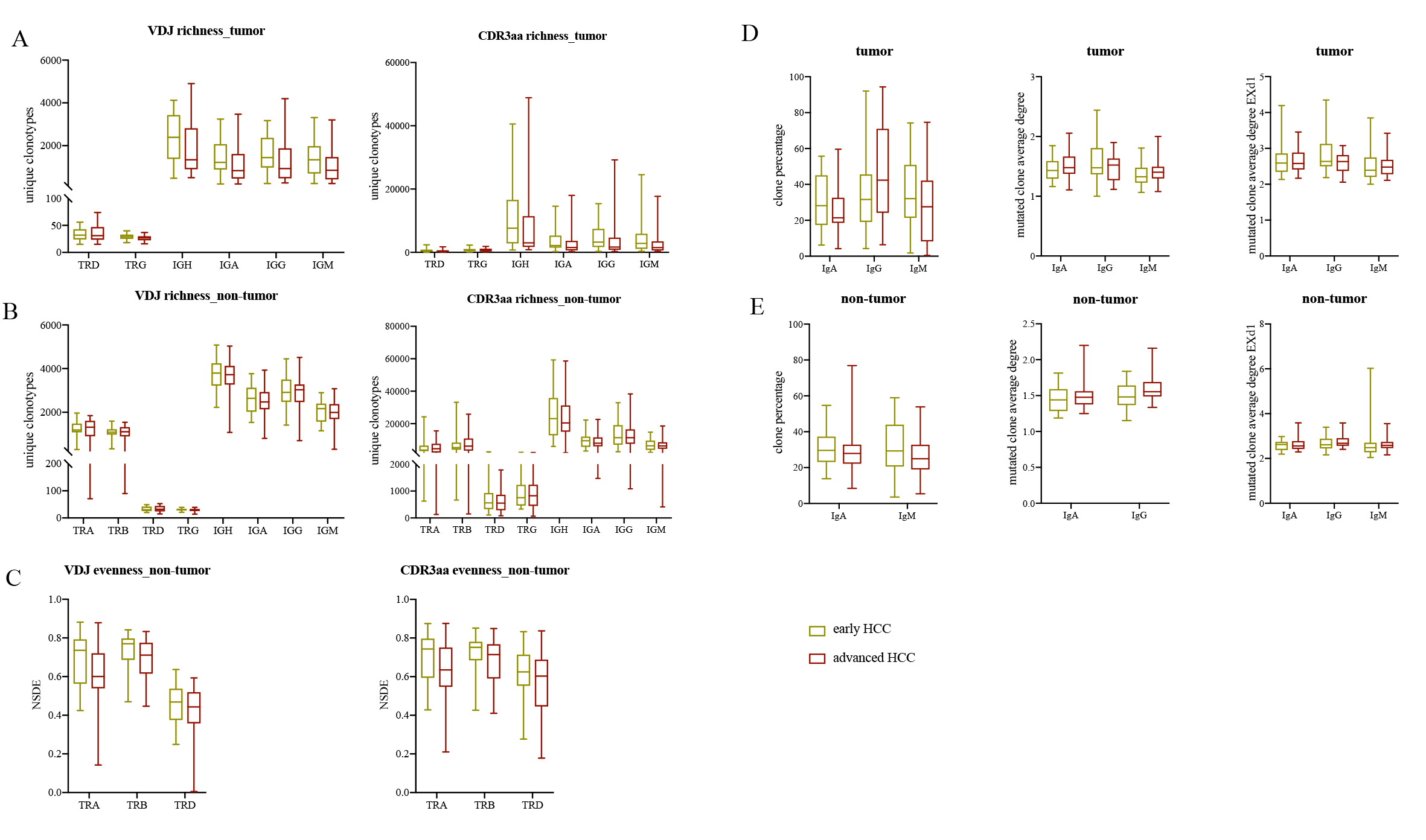

Supplement: Supplementary Figure 6 — Differences in IR and BCR SHM between early- and advanced-stage HCC. (A) IR richness in tumor tissues. (B) IR richness in adjacent non-tumor tissues. (C) IR evenness in non-tumor tissues. BCR SHM differences in tumor (D) and adjacent non-tumor tissues (E). green: early-stage HCC, red: advanced-stage HCC. BCR, B cell receptor; HCC, hepatocellular carcinoma; IR, immune repertoire; SHM, somatic hypermutation. [file Image_6.tif]

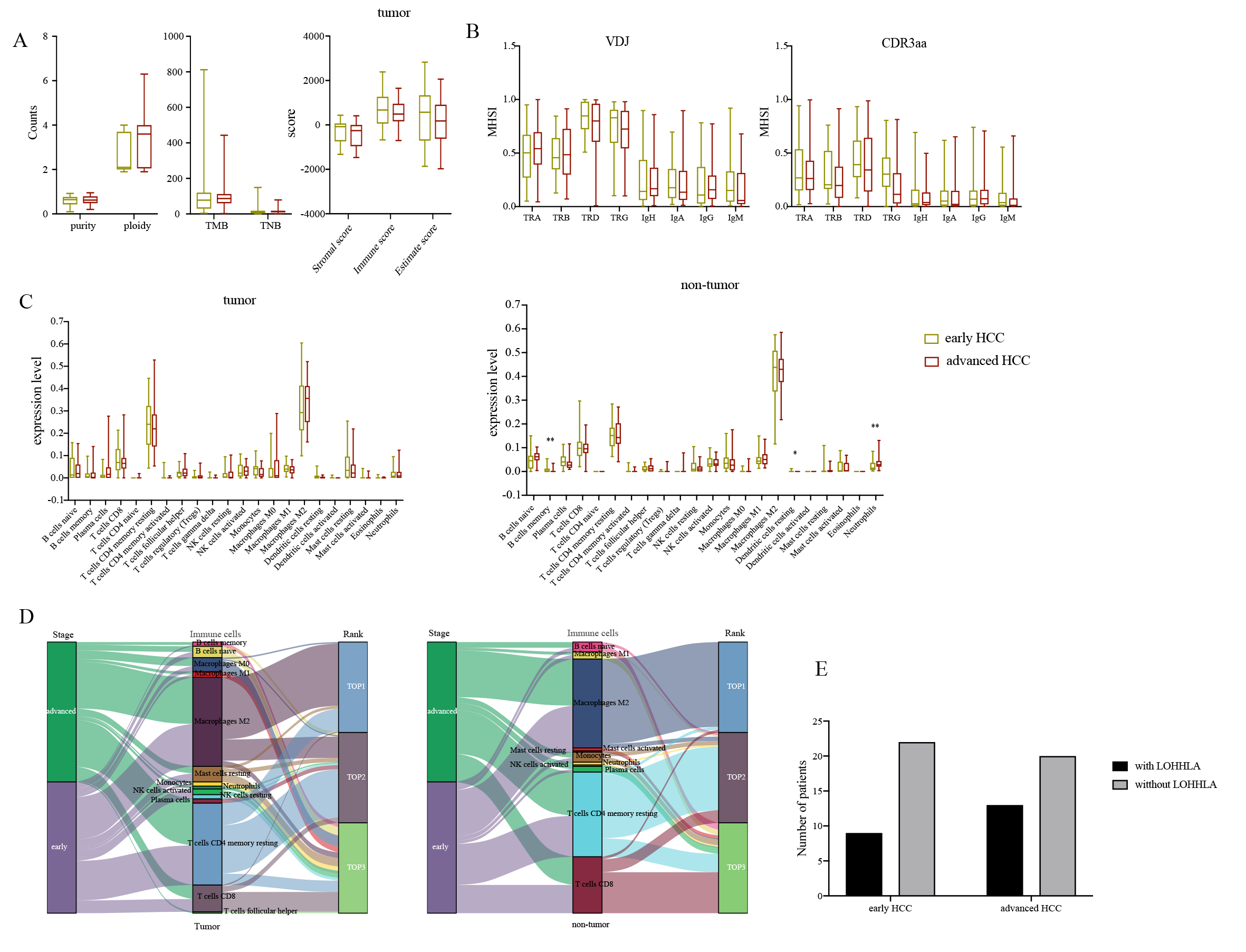

Supplement: Supplementary Figure 7 — Difference in immune infiltration between early- and advanced-stage HCC. (A) Tumor molecular and stromal score, immune score and estimate score. (B) MHSI. Immune cell infiltration in tumor and adjacent non-tumor tissues (C, D) green: early-stage HCC, red: advanced-stage HCC. (E) Frequency of HLA LOH at different stages of HCC. Left: early-stage HCC, right: advanced-stage HCC; grey: without HLA LOH; black: with HLA LOH. HCC, hepatocellular carcinoma; HLA, human leukocyte antigen; LOH, loss of heterozygosity; MHSI, Morisita–Horn similarity index. [file Image_7.tif]

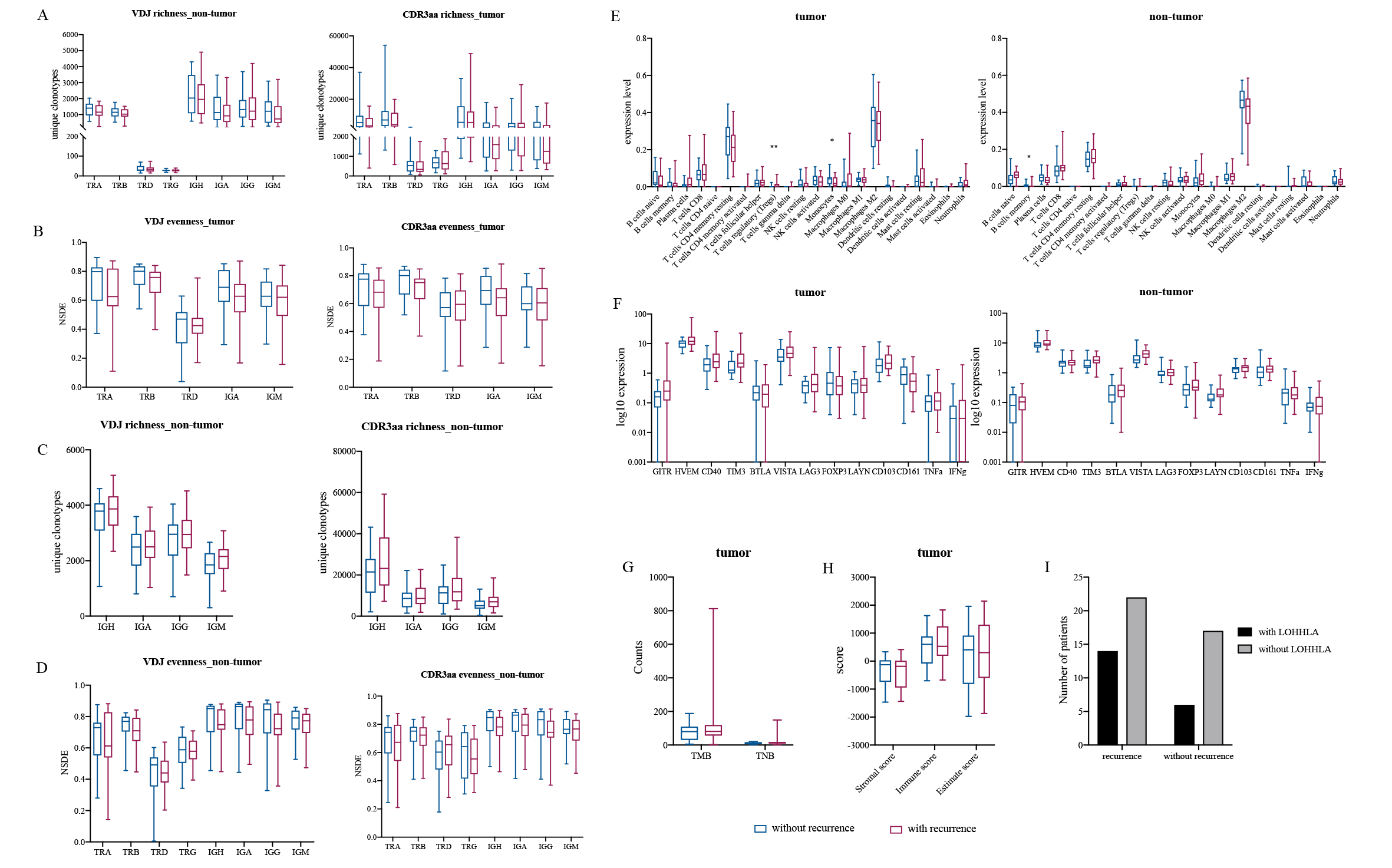

Supplement: Supplementary Figure 8 — Differences in IR and molecular features between HCC patients with and without recurrence. (A) IR richness in tumor tissues. (B) IR evenness in tumor tissues. (C) IR richness in non-tumor tissues. (D) IR evenness in non-tumor tissues. (E) Immune cells infiltration in tumor and non-tumor tissues. (F) Checkpoint expression in tumor and non-tumor tissues. (G) Molecular features of tumors. (H) Immune score in tumor tissues. blue: without recurrence; red: with recurrence. (I) LOH in HLA. Left: without recurrence, right: with recurrence; grey: without HLA LOH; black: with HLA LOH. HCC, hepatocellular carcinoma; HLA, human leukocyte antigen; IR, immune repertoire; LOH, loss of heterozygosity. [file Image_8.tif]

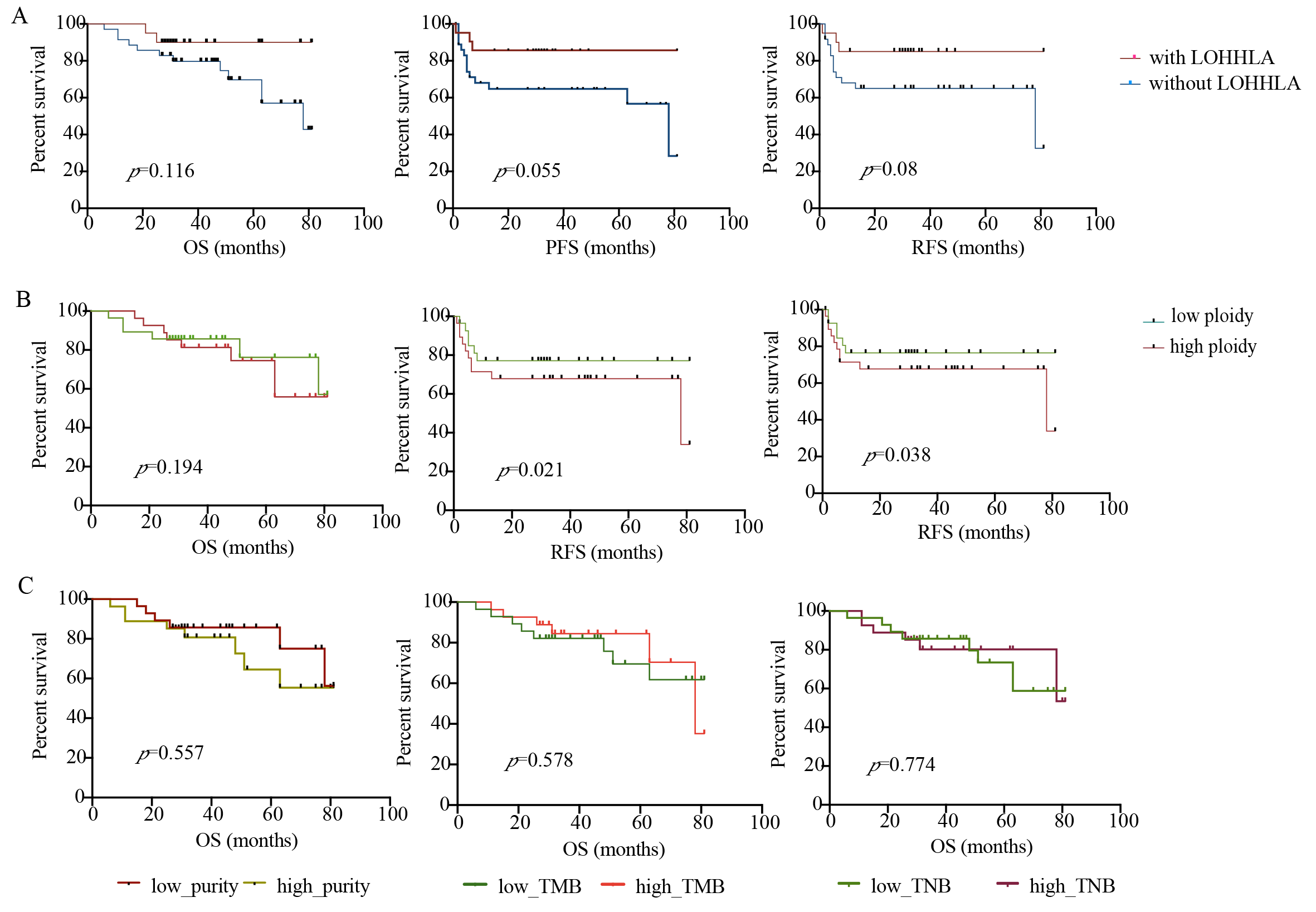

Supplement: Supplementary Figure 9 — Survival analysis based on HLA LOH, tumor ploidy, TMB, and TNB in HCC. (A) Better survival in patients with HLA LOH. (B) Longer RFS in patients with low tumor ploidy. (C) No significant difference between high TMB/TNB and low TMB/TNB. HCC, hepatocellular carcinoma; HLA, human leukocyte antigen; LOH, loss of heterozygosity; RFS, recurrence-free survival; TMB, tumor mutation burden; TNB, tumor neoantigen burden. [file Image_9.tif]
